# Patient considerations for orthodontists: A comparative study of university students in Malaysia and Taiwan

**DOI:** 10.1016/j.jds.2024.07.003

**Published:** 2024-07-17

**Authors:** Tee Chi Yuo, Johnson Hsin-Chung Cheng, Daniel De-Shing Chen, Renu Sarah Samson, Eby Varghese, Od Bayarsaikhan

**Affiliations:** a School of Dentistry, College of Oral Medicine, Taipei Medical University, Taipei City, Taiwan; b Orthodontic Division, Department of Dentistry, Taipei Medical University Hospital, Taipei City, Taiwan; c Department of Orthodontics, Manipal University College Malaysia, Malacca, Malaysia; d Department of Pediatric Dentistry, Manipal University College Malaysia, Malacca, Malaysia; e Department of Orthodontics, School of Dentistry, Mongolian National University of Medical Sciences, Ulaanbaatar, Mongolia

**Keywords:** Patients' consideration, Patients' preference, Orthodontist, Malaysia, Taiwan

## Abstract

**Background/purpose:**

Patients' considerations when choosing an orthodontist are influenced by many factors, including background, ethnicity, and location. Accordingly, this study aimed to identify factors influencing patients’ considerations when selecting an orthodontist in both Malaysia and Taiwan.

**Materials and methods:**

In total, 248 dental students from Taipei Medical University and 110 dental students from Manipal University College Malaysia were selected for this study. Participants' considerations when selecting an orthodontist were assessed using a questionnaire survey. The questionnaire collected data regarding participants’ demographic characteristics and their preferences regarding clinical settings, orthodontist attributes, administrative systems, and the influence of social media. The gathered data were analyzed and compared using independent *t*-test, ANOVA, and chi-squared for both cohorts.

**Results:**

The present results revealed significant differences between the Malaysian and Taiwanese participants with several variables, including orthodontist experience, recommendations, pain-free treatment procedures, treatment duration, friendly reception, sources of information about orthodontists, and preferred social media platforms. Notably, among the Taiwanese participants, “person responsible for treatment costs,” was significantly correlated with the orthodontist's age, the orthodontist's work experience, information sources, travel distance, and content posted by orthodontists on social media. By contrast, among the Malaysian participants, this variable was correlated with the work experience of orthodontists.

**Conclusion:**

Significant differences were observed between the Malaysian and Taiwanese participants in terms of their considerations when choosing an orthodontist. Participant's gender significantly influenced orthodontist preferences among the Malaysian participants, whereas the individual responsible for treatment costs was identified to be the most crucial factor influencing the Taiwanese participants.

## Introduction

Orthodontists are the primary providers of orthodontic treatment.^1^ Therefore, orthodontists should understand the preferences of patients in the selection of an orthodontist. Demographic variables play a notable role in patients’ choices of orthodontists. For example, a study conducted in India reported a considerable influence of orthodontist demographics on patient preferences.^2^ By contrast, in the United States, empathy and ethical standards have been identified as essential criteria for dental provider selection.^3^^,^^4^

A study conducted in Romania revealed competence as the primary factor influencing patients' selection of dentists.^5^ Moreover, nearly half of a sample of Canadian patients (49.4%) preferred orthodontic specialists for teeth alignment.^6^ Patients in Saudi Arabia tended to choose dentists with established reputations.^7^ These research findings highlight the diverse array of factors that influence patients’ considerations when choosing an orthodontist.

In addition, patients' preferences for orthodontists are subject to change according to their cultural background and geographical location. Furthermore, patients’ ethnicity can influence their choices when seeking an orthodontist. However, no study has specifically explored patient preferences in selecting an orthodontist in Eastern countries. Therefore, the main objective of this study was to specifically identify patient considerations when selecting orthodontists in Malaysia and Taiwan.

## Materials and methods

The present study protocol was approved by the Institutional Review Board of Taipei Medical University Hospital (approval no.: N202306063) and Manipal University College Malaysia (reference no.: 051/2023).

This study conducted an anonymous academic questionnaire survey. The questionnaire was collaboratively developed by three researchers, namely the coauthor and two other authors. On the basis of a review of more than 50 articles, the authors developed a questionnaire that aligned closely with the present research topic. This questionnaire was designed to assess participants’ preferences for orthodontist selection across multiple domains, including the clinical setting, orthodontist attributes, administrative system, and the influence of social media. Every item in the questionnaire was derived from at least one previous study.

The questionnaire comprised three sections. The first section, comprising five questions, gathered the demographic characteristics of the participants (Table 1). The second section comprised eight multiple-choice questions (Table 2). The third section comprised 22 cognitive questions, with a visual analog scale (VAS) used for assessments (Table 3). The participants were instructed to rate their preferences regarding orthodontist selection on a 100-mm VAS.^8^, ^9^, ^10^

**Table 1:** Demographic characteristics.

Factor	Variable	Taiwan		Malaysia		Chi-squared test
		Frequency	Percentage (%)	Frequency	Percentage (%)	*P*
Gender	Male	132	53.2	28	25.5	0.654
	Female	116	46.4	82	74.5	
Person responsible for orthodontic treatment costs	Self	51	20.6	12	10.9	0.921
	Parents	192	77.4	96	87.3	
	Others	5	2	2	1.8	
Self-assessment of orthodontic knowledge	High	124	50	59	10.9	0.475
	Moderate	118	47.6	86	78.2	
	Low	6	2.4	12	10.9	
Experience of orthodontic treatment	Yes	107	43.1	59	53.6	0.714
	No	141	56.9	51	46.4	
Frequency of dental check-ups	Once every 3 months	26	10.5	20	18.2	0.063
	Once every 6 months	173	69.8	49	44.5	
	Once a year	34	13.7	36	32.7	
	Less than once a year	15	6	5	4.5	

*P* < 0.05.

**Table 2:** Frequency distribution of participants’ preferences.

Factor	Variable	Taiwan		Malaysia		Chi-squared test
		Frequency	Percentage (%)	Frequency	Percentage (%)	*P*
Age of the orthodontist (years)	25–35	20	8.1	9	8.2	0.21
	35–45	143	57.7	55	50	
	45–55	75	30.2	38	34.5	
	55–65	6	2.4	7	6.4	
	>65	4	1.6	1	0.9	
Orthodontic work experience (years)	1–5	4	1.6	7	6.4	0.589
	5–10	71	28.6	54	49.1	
	11–15	133	53.6	36	32.7	
	16–20	25	10.1	10	9.1	
	≥21	15	6	3	2.7	
Sources of receiving information about orthodontists	Social media	36	14.5	14	12.7	0.034^a^
	Friends	58	23.4	25	22.7	
	Family members	74	29.8	43	39.1	
	Other dentists	49	19.8	17	15.5	
	Internet	31	12.5	11	10	
Attire of the orthodontist	Scrubs	38	15.3	84	76.4	0.365
	White coat	197	79.4	8	7.3	
	Casual attire	13	5.2	18	16.4	
Maximum travel distance (by car) to the orthodontist's clinic (min)	10	25	10.1	21	19.1	0.958
	20	69	27.8	39	35.5	
	30	111	44.8	34	30.9	
	40	18	7.3	9	8.2	
	>40	25	10.1	7	6.4	
Response to differences regarding treatment plan with orthodontist	Complete trust in the orthodontist's recommendations	29	11.7	30	27.3	0.846
	Insistence on own preferences	4	2	7	6.4	
	Compromise after discussion with the orthodontist	215	86.3	73	65.5	
Preferred social media platforms	Instagram	202	81.5	78	70.9	0.001^a^
	Facebook	15	6	7	6.34	
	Other	31	12.5	25	22.7	
Type of preferred content posted by orthodontists on social media	Relationship building with the patient	32	12.9	2	1.8	0.985
	Information about orthodontic treatments	137	55.2	49	44.5	
	Entertainment only	9	3.6	2	1.8	
	Before and after photographs of orthodontic treatment cases	43	17.3	50	5.5	
	Awareness posts regarding oral hygiene	24	9.7	5	4.5	
	Others	3	1.2	2	1.8	

*P* < 0.05

a Indicates a significant difference.

**Table 3:** Correlations between participant preferences and orthodontist selection.

Factor	Taiwan		Malaysia		*t* test
	Mean	SD	Mean	SD	*P*
Age of the orthodontist	4.76	2.16	4.61	2.03	0.531
Overall reputation of the orthodontist	7.7	1.6	7.66	1.43	0.775
Orthodontist's experience	8.5	1.36	8	1.3	0.001^a^
Friendly and caring attitude of the orthodontist	7.6	1.76	7.89	1.58	0.320
Communication skills of the orthodontist	7.9	1.55	8.07	1.54	0.352
Word of mouth/recommendations from family members or friends	7.59	1.63	6.16	2	0.001^a^
Skills and knowledge of the orthodontist	8.6	1.19	8.74	1.04	0.650
Pain-free treatment procedures	6.23	2.3	7	2	0.001^a^
Advanced equipment use	6.86	1.83	6.68	1.8	0.390
Cleanliness and hygiene of clinic	8.4	1.37	8.55	1.2	0.325
Clinic decor	5.87	2.03	4.87	2.43	0.001^a^
Patient privacy during treatment	7.94	1.58	7.79	1.79	0.438
Friendliness of nurses	7.4	1.92	7.69	1.75	0.172
Friendly reception	6.11	2.13	7.55	1.8	0.001^a^
Telephone reminders about orthodontic appointments	6.4	2.19	7.5	1.92	0.001^a^
Online appointment system	6.12	2.32	6.45	2.33	0.216
Effectiveness of social media advertising	5.69	5.2	5.62	2.49	0.878
Online reviews about the orthodontist	6.98	1.84	6.9	2.03	0.686
Engagement of new patients through social media	5.53	2.33	5.05	2.29	0.068
Treatment costs	7.45	1.8	7.78	1.75	0.117
Access to orthodontic emergency treatment at any time	7.24	1.88	7.65	1.75	0.058
Total treatment duration	6.72	1.99	7.2	2.16	0.041^a^

a Indicates a positive correlation; SD = standard deviation.

The reliability of the questionnaire was assessed by administering a pretest and kappa test. The participants were categorized by nationality (Malaysian and Taiwanese), with a separate kappa test conducted for each group. The kappa tests yielded results of 0.70 for the Malaysian participants and 0.75 for the Taiwanese participants, indicating substantial agreement in both countries.

Following the pretest, dental students were randomly selected from the dental departments of Taipei Medical University (Taiwan) and Manipal University College Malaysia (Malaysia). Prior to data collection, the participants received a comprehensive introduction to the research. Subsequently, informed consent was obtained from each participant. Data were collected only from participants who had voluntarily agreed to participate in this study and had signed the consent form.

Two weeks following the completion of questionnaire collection, a subset of 35 participants (10% of the total) was randomly selected for test–retest analysis to evaluate the reliability of the questionnaire. The resulting reliability coefficient for the questionnaire was 0.88.

Statistical analyses were performed using SPSS Statistics (version 19.0, SPSS, Chicago, IL, USA). Differences in demographic characteristics and orthodontist selection preferences between the Malaysian and Taiwanese participants were evaluated using a chi-squared test and independent *t*-test. The correlation between each participant's preference and demographic traits was evaluated using a *t*-test, ANOVA, and a chi-squared test. The significance level was set at a *P*-value <0.05.

## Results

### Demographic characteristics of the participants

A total of 358 dental students participated in this study, namely 248 from Taiwan and 110 from Malaysia. No significant differences in demographic characteristics were observed between the Malaysian and Taiwanese participants (Table 1).

## Results derived from multiple-choice questions assessments

Table 2 indicates significant differences in the sources of information about orthodontists and the platforms of social media preferred by the Malaysian and Taiwanese participants (*P* < 0.05). Substantial proportions of the participants from Taiwan and Malaysia (29.8% and 39.1%, respectively) received orthodontist information from family members. Moreover, 81.5% and 70.9% of the Taiwanese and Malaysian participants, respectively, primarily used Instagram during their free time (*P* < 0.001).

## Results derived from visual analog scale assessments

Table 3 reveals a notable finding: The participants from both countries preferred an orthodontist with high levels of skill and knowledge and abundant experience. However, they did not consider the orthodontist's age to be a crucial factor, despite the assumption that older orthodontists may possess greater skill and likely have more experience.

Table 3 reveals significant differences (*P* < 0.01) between the Taiwanese and Malaysian participants related to orthodontist experience, recommendations of orthodontists, pain-free treatment procedures, clinic decoration, friendly reception, and telephone reminders.

### Correlations between demographic factors and participant preferences

Table 4 highlights significant differences among the Malaysian participants regarding participant's gender and sources of received orthodontist information. By contrast, among the Taiwanese participants, significant differences were noted regarding gender and the type of content that orthodontists posted on social media. Moreover, the Malaysian participants exhibited significant differences in the person responsible for orthodontic treatment costs and years of orthodontist experience (*P* = 0.047). By contrast, the Taiwanese participants exhibited a significant difference only in the correlation between their preferences and the person responsible for the cost of orthodontic treatment, as shown in Table 4.

**Table 4:** Relationship between demographic characters and preferences between the Malaysian and Taiwanese participants.

	Gender		Person responsible for orthodontic treatment costs		Self-assessment of orthodontic knowledge		Experience of orthodontic treatment		Frequency of dental check-ups	
	Malaysia	Taiwan	Malaysia	Taiwan	Malaysia	Taiwan	Malaysia	Taiwan	Malaysia	Taiwan
Age of the orthodontist	0.104	0.948	0.357	0.001^a^	0.957	0.05	0.347	0.186	0.465	0.05
Years of orthodontic work experience	0.534	0.955	0.047^a^	0.001^a^	0.794	0.269	0.21	0.756	0.505	0.273
Sources of receiving information about orthodontists	0.023^a^	0.117	0.671	0.023^a^	0.523	0.959	0.118	0.001^a^	0.029	0.331
Maximum travel distance (by car) to the orthodontist's clinic	0.182	0.481	0.631	0.001^a^	0.401	0.083	0.927	0.468	0.096	0.168
Type of preferred content posted by orthodontists on social media	0.092	0.026^a^	0.359	0.043^a^	0.536	0.264	0.474	0.002^a^	0.832	0.398

P < 0.05

a Indicates a significant difference.

### Correlation between demographics and participant preferences

As depicted in Table 5, the correlations of considerations in orthodontist selection with gender, person responsible for orthodontic treatment costs, experience of an orthodontist, and frequency of dental check-ups significantly differed between the Malaysian and Taiwanese participants. Conversely, the correlation between the participants’ self-assessed knowledge of orthodontic treatment and their preferences did not significantly differ between the Malaysian and Taiwanese participants (*P* < 0.05). Table 4, Table 5 show that in Malaysian participants, there are significant differences between gender and 6 participant preferences. However, among Taiwanese participants, gender only has a significant difference with 3 participant preferences. This demonstrates that the gender of the participant has a greater impact on Malaysian participants compared to Taiwanese participants.

**Table 5:** Relationship between demographic factors and preferences between the Malaysian and Taiwanese participants.

Factor	Gender		Person responsible for orthodontic treatment costs		Self-assessment of orthodontic knowledge		Experience of orthodontic treatment		Frequency of dental check-ups	
	Malaysia	Taiwan	Malaysia	Taiwan	Malaysia	Taiwan	Malaysia	Taiwan	Malaysia	Taiwan
Overall orthodontist reputation	0.583	0.194	0.292	0.922	0.969	0.561	0.92	0.255	0.987	0.05
Orthodontist's experience	0.767	0.03^a^	0.326	0.246	0.862	0.588	0.145	0.413	0.12	0.12
Friendly and caring attitude of the orthodontist	0.038^a^	0.678	0.067	0.22	0.301	0.216	0.839	0.285	0.279	0.909
Communication skills of the orthodontist	0.04^a^	0.441	0.487	0.284	0.532	0.388	0.76	0.473	0.049^a^	0.028^a^
Word of mouth/recommendations from family members or friends	0.590	0.004^a^	0.291	0.281	0.464	0.416	0.711	0.044^a^	0.214	0.213
Skills and knowledge of the orthodontist	0.137	0.079	0.041^a^	0.687	0.33	0.965	0.292	0.329	0.267	0.2
Pain-free treatment procedures	(−)	(−)	(−)	(−)	(−)	(−)	(−)	(−)	(−)	0.043^a^
Clinic decor	0.483	0.821	0.14	0.036^a^	0.996	0.230	0.945	0.021^a^	0.967	0.337
Patient privacy during treatment	0.964	0.975	0.963	0.466	0.406	0.649	0.746	0.049^a^	0.846	0.997
Friendliness of nurses	0.001^a^	0.197	(−)	(−)	(−)	(−)	(−)	(−)	(−)	(−)
Friendly reception	0.022^a^	0.215	(−)	(−)	(−)	(−)	0.046^a^	0.726	(−)	(−)
Effective social media advertising	0.616	0.694	0.519	0.817	0.548	0.395	0.17	0.351	0.501	0.026^a^
Engagement of new patients through social media	0.277	0.015^a^	0.026^a^	0.152	0.689	0.226	0.089	0.115	0.147	0.016
Treatment costs	0.007^a^	0.26	(−)	(−)	(−)	(−)	(−)	(−)	(−)	(−)

*P* < 0.05

a Indicates a significant difference; (−) indicates a nonsignificant different.

## Discussion

As mentioned, this study employed a VAS to evaluate the characteristics of orthodontists preferred by the participants. A VAS was selected for this study because it is a valid and reliable method for assessing participants' levels of pain, preferences, attitudes, and feelings.^8^, ^9^, ^10^ Moreover, such scales are known to yield more accurate results than multiple-choice questions; thus, they tend to lead to a more detailed and precise understanding of participants’ considerations when selecting an orthodontist.

This study also examined the influence of demographic factors on the participants’ preferences. The Malaysian and Taiwanese participants exhibited different outcomes on the basis of their nationalities. No other study has yet compared patient preferences for orthodontists between these two countries; in other words, this study was the first to examine the impact of demographic information on patient preferences for orthodontists across these two nationalities.

The results in Table 3 indicate that clinic decor was not a vital criterion influencing the Taiwanese and Malaysian participants. Among the Malaysian participants, no significant difference (*P* > 0.05) was noted between clinic decor and demographic factors, indicating that the clinic environment was not a crucial factor affecting their selection of an orthodontist.^11^ Many studies^5^^,^^7^^,^^11^^,^^12^ have highlighted that a clinic's color scheme, environment, and decor are not crucial factors considered by patients in selecting a dentist, possibly because patients tend to prioritize an orthodontist's skills and knowledge over the clinic's aesthetics. However, this finding contrasts with a study conducted in Pakistan,^13^ which revealed that patients with different demographic backgrounds may have different preferences when selecting an orthodontist.

Furthermore, clinic cleanliness was highly prioritized by both the Malaysian and Taiwanese participants in the present study. This finding was consistent with the results of a study conducted by Tugce,^14^ which indicated that although a clinic does not necessarily require extravagant decor, its cleanliness must be maintained.

The results presented in Table 4 indicate no correlation between demographic factors and maximum travel distance among the Malaysian participants, suggesting that under certain circumstances, the location of a dental clinic does not significantly influence participants’ decisions when choosing a dentist.^12^^,^^15^

Both the Malaysian and Taiwanese participants considered the orthodontist's knowledge to be the most crucial factor influencing orthodontist selection (Table 3). This finding aligns with those of a study conducted by Mustafa Ersöz.^16^ Similar preferences have been observed for the selection of general dentists; specifically, participants in previous studies have prioritized dentist competence and overall treatment quality.^5^^,^^17^ Notably, despite the high value placed on orthodontists' knowledge and experience,^13^ the age of the orthodontist was rated as the least important criterion among both the Malaysian and Taiwanese participants in the present study (Table 3). This finding is consistent with the study conducted by Souza-Constantino et al., where adolescent participants also did not consider the age of the orthodontist to be a significant factor.^18^ This may be because participants believe that younger orthodontists are more likely to employ the latest technology in their treatments. However, because orthodontic treatment requires considerable experience, the age of the orthodontist should not be overlooked.

This study revealed that individuals with experience of undergoing orthodontic treatment consider recommendations from family members and friends to be important. Similarly, previous studies have indicated that recommendations from family members, friends, and other dentists significantly influence patients' decisions regarding orthodontist selection.^19^^,^^20^ As indicated in Table 3, in the present study, no significant difference was observed between the Malaysian and Taiwanese participants for the friendly and caring attitude of the orthodontist. However, many studies have emphasized that an orthodontist's caring attitude plays a crucial role in patients' selection preferences.^11^^,^^21^

No significant differences were observed for demographic characteristics with orthodontic treatment costs between the participants from Malaysia and Taiwan except with gender among the Malaysian participants (*P* < 0.01). This discrepancy may be because most of the participants' parents were responsible for orthodontic treatment costs; this factor was considered moderately important according to the VAS assessments. This finding contrasts with many studies that have indicated that the cost of dental treatment significantly influences patients’ willingness to undergo such treatment.^20^^,^^22^, ^23^, ^24^ The present findings suggested that when treatment cost was not a primary concern, the participants tended to focus more on orthodontist characteristics, such as experience, knowledge, and communication skills, as well as clinic cleanliness (Table 3).

Many studies related to dentistry and medicine have explored the use of electronic media and patient engagement with social media.^25^, ^26^, ^27^, ^28^, ^29^ However, the influence of media on patient decision-making remains unclear.^11^^,^^30^ In Malaysia, a minority of orthodontists incorporate social media into their marketing strategies.^31^ Notably, both the Malaysian and Taiwanese participants in this study ranked effective social media advertising and engagement of new patients through social media among the least important criteria for orthodontist selection (Table 3). The mean VAS scores for the importance of social media advertisements were 5.69 among the Taiwanese participants and 5.62 among the Malaysian participants, ranking this variable 20th and 19th, respectively, of 22 items related to the participants’ preferences in the VAS assessment. Similarly, the mean VAS scores for engagement of new patients through social media were 5.53 among the Taiwanese participants and 5.05 among the Malaysian participants, ranking this variable 21st and 20th, respectively. These findings are consistent with those of a study indicating that people are generally more inclined to trust personal sources over impersonal sources.^20^ However, other studies have suggested that online marketing strategies can significantly influence the engagement of new patients,^32^^,^^33^ particularly female patients.^34^

This study had several limitations that should be acknowledged. First, the target sample was limited to dental students, which may have led to bias. Second, because this study primarily recruited young participants, its findings may not be generalizable to the broader population. Therefore, future research should incorporate more extensive and diverse samples spanning wider age ranges; this approach would enhance the validity and representativeness of the present study's findings.

This study reveals a 33% significant difference in participant preferences between Malaysian and Taiwanese participants. Significant differences (*P* < 0.01) were observed between Malaysian and Taiwanese participants in the type of social media platform preferred by the participants, recommendations of orthodontists, clinic decoration, and friendly reception. Furthermore, gender of the participants significantly influenced orthodontist preferences among the Malaysian participants, whereas the individual responsible for treatment costs was identified to be the most crucial factor influencing the Taiwanese participants. These findings underscore the influential role of cultural background in patients' decision-making processes. Therefore, a similar study should be conducted in different countries to compare and identify the patient's preferences in those countries. The result of this study could offer valuable guidance related to clinical management for orthodontists and thus could contribute to enhancing the quality of their practice in Malaysia and Taiwan.

## Declaration of competing interest

The authors have no conflicts of interest relevant to this article.
